# Modular Construction of Prussian Blue Analog and TiO_2_ Dual‐Compartment Janus Nanoreactor for Efficient Photocatalytic Water Splitting

**DOI:** 10.1002/advs.202001987

**Published:** 2021-02-16

**Authors:** Chunjing Shi, Sheng Ye, Xuewen Wang, Fanning Meng, Junxue Liu, Ting Yang, Wei Zhang, Jiatong Wei, Na Ta, Gao Qing (Max) Lu, Ming Hu, Jian Liu

**Affiliations:** ^1^ State Key Laboratory of Catalysis Dalian Institute of Chemical Physics Chinese Academy of Sciences, and Dalian National Laboratory for Clean Energy 457 Zhongshan Road Dalian 116023 P. R. China; ^2^ School of Physics and Materials Science East China Normal University 500 Dongchuan Road Shanghai 200241 P. R. China; ^3^ The College of Chemistry Nanchang University 999 Xuefu Road Nanchang 330031 P. R. China; ^4^ DICP‐Surrey Joint Centre for Future Materials Department of Chemical and Process Engineering University of Surrey Guildford Surrey GU2 7XH UK; ^5^ University of Surrey Guildford Surrey GU2 7XH UK

**Keywords:** bifunctional water splitting, Janus nanoreactor, Prussian blue analog, TiO_2_

## Abstract

Janus structures that include different functional compartments have attracted significant attention due to their specific properties in a diverse range of applications. However, it remains challenge to develop an effective strategy for achieving strong interfacial interaction. Herein, a Janus nanoreactor consisting of TiO_2_ 2D nanocrystals integrated with Prussian blue analog (PBA) single crystals is proposed and synthesized by mimicking the planting process. In situ etching of PBA particles induces nucleation and growth of TiO_2_ nanoflakes onto the concave surface of PBA particles, and thus enhances the interlayer interaction. The anisotropic PBA–TiO_2_ Janus nanoreactor demonstrates enhanced photocatalytic activities for both water reduction and oxidation reactions compared with TiO_2_ and PBA alone. As far as it is known, this is the first PBA‐based composite that serves as a bifunctional photocatalyst for solar water splitting. The interfacial structure between two materials is vital for charge separation and transfer based on the spectroscopic studies. These results shed light on the elaborate construction of Janus nanoreactor, highlighting the important role of interfacial design at the microscale level.

## Graphical abstract

The 2D nanocrystals can grow on etched faces of metal–organic framework (MOF) single crystals to form Janus nanoreactor. The rational interface engineering of Janus nanoreactor is the key to realizing efficient photocatalytic water reduction and oxidation reactions. This work opens up a new window for the design and construction of Janus nanoreactor as promising heterojunction photocatalysts.

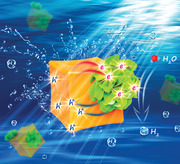

## 1 Introduction

Janus structures, consisting of two joined components that can be designed differently and individually to achieve a desirable characteristics in shape, composition, chemistry, polarity, functionality, electrical, and other properties.^[^ ^1^ ^]^ Up to now, various strategies have been reported for the synthesis of Janus particles, such as phase separation,^[^ ^2^ ^]^ self‐assembly,^[^ ^3^ ^]^ surface nucleation, seeded growth,^[^ ^4^ ^]^ microfluidics,^[^ ^5^ ^]^ and Pickering emulsions interfacial synthesis,^[^ ^6^ ^]^ etc. These strategies allow the fabrication of multifunctional materials and thus endow Janus particles with very unique properties.^[^ ^7^ ^]^ Such anisotropic materials have attracted increasing attention in recent years because of their great potential in a range of applications, including interfacial stabilizers, sensors,^[^ ^8^ ^]^ drug delivery,^[^ ^9^ ^]^ optics,^[^ ^10^ ^]^ and catalysis.^[^ ^11^ ^]^ Notably, production of hydrogen and oxygen through photocatalytic water splitting process is very promising process for solar energy utilization.^[^ ^12^ ^]^

2D nanosheets have high specific surface area, abundant catalytic active sites, and shorter diffusion length of charge carriers, thus stimulating a wide range of interests in photocatalytic water splitting.^[^ ^13^ ^]^ Meanwhile, the absence of grain boundaries and nanocrystalline domains, makes single crystals the ideal platform to probe the intrinsic material properties as well as the surface recombination.^[^ ^14^ ^]^ Therefore, it is desirable to develop efficient photocatalysts composed of 2D nanocrystals and porous single crystals with desired shape and surface chemistry. Nevertheless, there are still many obstacles due to the difficulty in manipulating a huge number of microparticles for spatially selective surface modification. First, the adhesion between 2D nanocrystals and porous single crystals are relatively weak if the two crystals are not bonded through epitaxial growth. Second, the 2D nanocrystals tend to stack on the surface of single crystals if free‐standing structure cannot be realized. Thus, it is very significant to employ the strategy of in situ etching and nucleation for designing a Janus nanoreactor comprising two hemistructures with 2D nanocrystals (semiconductor) and porous single crystals (MOF). Although the electrodeposition and adsorption methods were reported to construct the PBA–TiO_2_ composite, it still remains challenge to directly grow guest on single crystal particles through chemical bonds.^[^ ^15^ ^]^

Herein, the PBA–TiO_2_ Janus nanoreactor was constructed by mimicking the process of planting to allow self‐supported 2D semiconductor nanoflakes (TiO_2_) to grow on void surface of Prussian blue analogs (PBA), a type of metal organic frameworks (MOFs). In comparison with bare TiO_2_ and PBA, the PBA–TiO_2_ nanoreactor exhibits clearly improved photocatalytic activities for both water reduction and oxidation reactions. Specifically, the interfacial structure between two materials plays a crucial role in charge separation and transfer, confirmed by a variety of characterization method, such as UV–vis, photoluminescence (PL), electrochemical impedance spectroscopy (EIS), and surface photovoltage (SPV) spectra.

## 2 Results and Discussion

**Figure 1:**
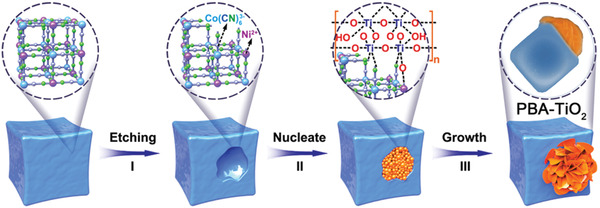
Schematic illustration of the synthetic strategy for the PBA–TiO_2_ Janus nanoreactor.

The schematic representation in **Figure** **1** illustrates our general concept for the formation of PBA–TiO_2_ Janus nanoreactor. In the present synthesis strategy, three key steps are involved. First, the partial dissociation of the PBA frameworks (Ni_3_[Co(CN)_6_]_2_) is induced by mild etching. It is noted that the protons play a key role in etching PBA crystals under hydrothermal/solvothermal conditions.^[^ ^16^ ^]^ Surface dissociation of the PBAs releases [Co(CN)_6_]^3−^ and Ni^2+^ ions, and makes unsaturated metal sites deposition onto the surface. Second, the unsaturated metal sites on the PBAs can form the chemical bond with the oxygen ions and Ti^4+^, inducing heterogenerous nucleation of the TiO*_x_* on the surface of the PBA single crystals. As a result, TiO*_x_* crystallites are supposed to take root in the PBA crystals. Lastly, the crystallites are expected to evolve into free‐standing flowers assembled by nanoflakes in the presence of ethylene glycol and glacial acetic acid as the morphology controlling agent.^[^ ^17^ ^]^ The morphology evolution of the products in each step is tracked as shown in Figure S1 in the Supporting Information.

The PBA cubes prepared via a precipitation method are used as the starting material.^[^ ^18^ ^]^ These particles are highly uniform with an average size of about 200 ± 40 nm (Figure S2a, Supporting Information). The crystallographic structure and phase purity of the PBA particles are examined by X‐ray diffraction (XRD) analysis. Clearly, all the diffraction peaks can be assigned to a face‐centered‐cubic (fcc) structure (*F*‐43*m* group) in line with typical Ni_3_[Co(CN)_6_]_2_ (JCPDS card no. 89‐3738) (Figure S2b, Supporting Information).^[^ ^19^ ^]^ These PBA crystals were dispersed in the solution of ethylene glycol and acetic acid to grow TiO_2_ on the surface. The PBA can be partially etched by acetic acid solution, while TiO_2_ is easy to nucleate in the solution. Without using the PBA seeds, the synthesized TiO_2_ crystals are anatase free‐standing flowers assembled with nanoflakes (Figure S3, Supporting Information).

**Figure 2:**
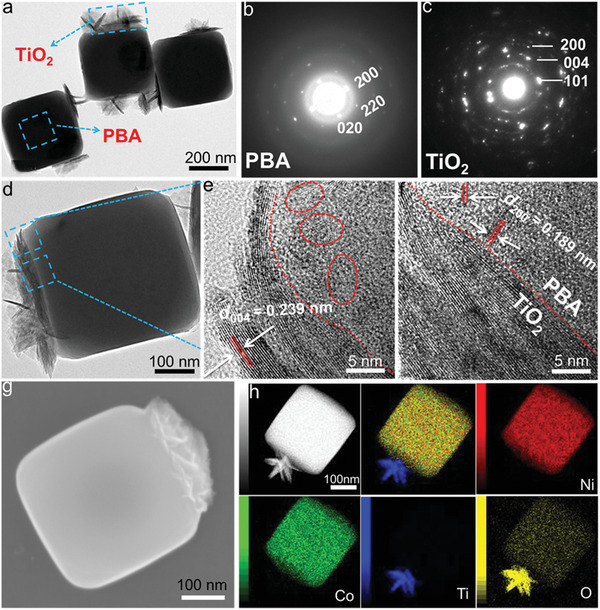
a,d) HRTEM images and b,c) SAED patterns of the PBA–TiO_2_ Janus particles. e,f) HRTEM image and g) SEM images of the obtained PBA–TiO_2_ Janus nanoreactors. h–m) The corresponding elemental mapping images of PBA–TiO_2_ Janus nanoreactors. The SEM and TEM images visually demonstrate the emergence of a Janus structure.

The morphologies and microstructure of the products are examined by scanning electron microscopy (SEM) and high‐resolution transmission electron microscopy (HRTEM). As shown in **Figure** **2**a and Figure S5a–c in the Supporting Information, the HRTEM images demonstrate that the PBA cubic structure is well preserved and the flowers‐like TiO_2_ are tightly grown onto the PBA cubes. As presented in Figure 2b, the selected‐area electron diffraction (SAED) pattern shows that the diffraction spots are arranged regularly, it indicates the single crystal PBA cube. The diffraction profile of the flower‐like structure is close to rings due to the different orientation of each grain, which can be assigned as anatase phase TiO_2_ (Figure 2c).^[^ ^20^ ^]^ HRTEM images illustrate that the nanoflakes are highly crystalline with a lattice spacing of around 0.23 and 0.18 nm corresponding to TiO_2_ in Figure 2d–f.^[^ ^21^ ^]^ Furthermore, Figure 2g and Figure S4 in the Supporting Information illustrate that flower‐like TiO_2_ are grown on the surface of the PBA cubes with uniform morphology. The image with high magnification illustrates that they are composed of nanoflakes. The PBA–TiO_2_ Janus nanoreactor contains a PBA cube and the TiO_2_ half flowers with the nanoflakes. In addition, Figure 2h–m gives high‐angle annular dark‐field (HAADF)‐scanning transmission electron microscope (STEM) images and elemental mapping of the particles in details. Ni and Co elements are evenly distributed in the cubes, while Ti and O elements are mainly found in TiO_2_ nanoflakes. In accordance with the above HRTEM and SEM observations, the results clearly suggest that a Janus structure composed of TiO_2_ flower and PBA crystal has been successfully prepared.

During the process of material preparation, the temperature, acidity and solvent have an important impact on the formation of the PBA–TiO_2_ Janus nanoreactor. When hydrochloric acid (1 mL 37% HCl) was used to replace the acetic acid, the PBA cubes were completely destroyed (Figure S6a, Supporting Information). This suggests that mild etching is necessary to form the PBA–TiO_2_ Janus particles. If the solvent ethylene glycol is exchanged with water, the products will not contain free‐standing TiO_2_ nanoflakes and only islands of TiO_2_ crystals exist on the surface of the PBA particles (Figure S6b, Supporting Information). Furthermore, the reaction temperature also plays a vital role. When the reactants were heated at 160 °C for 8 h, a small amount of 2D TiO_2_ nanoflakes were found on the surface of the PBAs cubes, covering one side of the PBA (Figure S6c, Supporting Information). Meanwhile, the SEM image of physical mixed PBA–TiO_2_ sample, as shown in Figure S6d in the Supporting Information, excludes the Janus phenomenon caused by physical mixing. These results demonstrate the importance of the ethylene glycol, acetic acid, and temperature toward producing Janus particles.

Moreover, the effect of titanium precursors concentration on the formation of Janus structure was further investigated. With an increase of the concentration of Ti^4+^ ions, it appears to be more assembled 2D nanoflakes although no significant difference in structure can be observed (Figure S7a–d, Supporting Information), which further proves the successful synthesis of the PBA–TiO_2_ Janus nanoreactor. Removal of the framework water in the PBA crystals is important for the formation of TiO_2_ flower‐like structures as well. The as‐prepared PBA (without being dried under vacuum) was used as the seeds to grow TiO_2_. Figure S8a in the Supporting Information shows that hemispheres are deposited on the PBA particles. The hemispherical structures are mixed anatase and rutile phase TiO_2_ crystals according to the powder X‐ray diffraction (PXRD) patterns (Figure S8b, Supporting Information). The probable reason is that the framework water may be created during the solvothermal process. Crystallization of TiO_2_ in the presence of too much water generally leads to a two‐phase structure.^[^ ^22^ ^]^

**Figure 3:**
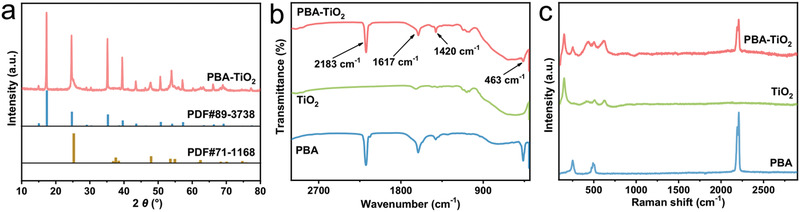
a) XRD pattern of PBA–TiO_2_ Janus nanoreactors. b) FTIR spectra and c) Raman spectra of PBA, TiO_2_, and PBA–TiO_2_ Janus nanoreactors.

The crystallographic structure and phase purity of the PBA–TiO_2_ Janus nanoreactor are examined by X‐ray powder diffraction (XRD), as shown in **Figure** **3**a. This analysis suggests that the resultant composite contains a face‐centered‐cubic (fcc) structure (*F‐43m* group) and a tetragonal structure (*I41* group) in line with PBA and TiO_2_, respectively. Besides, Fourier transform infrared spectroscopy (FTIR) spectrum of the PBA–TiO_2_ Janus nanoreactor displays a characteristic peak of 2183 cm^−1^, attributing to —CN vibration in PBA (Figure 3b). The intense absorption band at 463 cm^−1^ is ascribed to the stretching vibration of the Ni—CN bond.^[^ ^23^ ^]^ The absorption band at 1617 cm^−1^ is attributed to the stretching and bending vibrations of the hydroxyls in H_2_O between water molecules. Moreover, four Raman peaks appear at 147, 399, 519, and 642 cm^−1^, corresponding to typical Eg, B1g, B2g, and Eg vibrations of TiO_2_ (Figure S5d, Supporting Information).^[^ ^24^ ^]^ Figure 3c illustrates that CN telescopic vibration peaks were found at 2130 and 510 cm^−1^ in correspondence to the PBA. Based on the above results, the PBA–TiO_2_ composites were successfully prepared.

**Figure 4:**
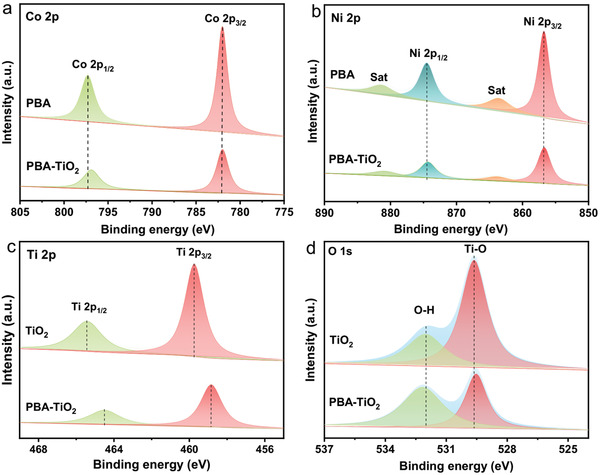
a) Co 2p XPS spectra and b) Ni 2p XPS spectra of the prepared PBA and PBA–TiO_2_ Janus nanoreactors, c) Ti 2p XPS spectra, and d) O 1s XPS spectra of the prepared TiO_2_ and PBA–TiO_2_ Janus nanoreactors.

In particular, X‐ray photoelectron spectroscopy (XPS) characterization confirms the existence of Ni, Co, Ti, and O peaks in PBA–TiO_2_ samples (Figure S9, Supporting Information). In the Co 2p spectrum (**Figure** **4**a), the binding energies of Co 2p_1/2_ shift from 782.0 to 797.3 eV in the PBA–TiO_2_ Janus particles.^[^ ^25^ ^]^ As can be seen from the Ni 2p spectra (Figure 4b), the peaks at 874.3 and 856.7 eV are assigned to the Ni^2+^ of the PBA and PBA–TiO_2_,^[^ ^26^ ^]^ while the two peaks are related to the shakeup satellite peak (Sat.).^[^ ^27^ ^]^ As shown in Figure 4c, the Ti 2p_1/2_ peak and 2p_2/3_ peak at the 465.4 and 459.8 eV shift to a lower binding energy of 464.4 and 458.8 eV, which is indicative of the incorporation of PBA into the TiO_2_ via chemical bonding.^[^ ^28^ ^]^ As seen in Figure 4d, the peak at 529.8 eV is the typical metal–oxygen bonds, and the peak at 532.2 eV corresponds to the water molecules adsorbed at the surface of PBA–TiO_2_, that show significant displacement compared to TiO_2_.^[^ ^29^ ^]^ These binding energy shifts provide circumstantial evidences of the interaction of Co(CN)_6_^3−^ and Ti^4+^ as well as Ni^2+^ and O^2−^. In fact, the tight growth of TiO_2_ on PBA cubes favors the formation of intimate interfacial contacts, which is highly favorable for efficient charge separation/transfer in the Janus nanoreactor.

**Figure 5:**
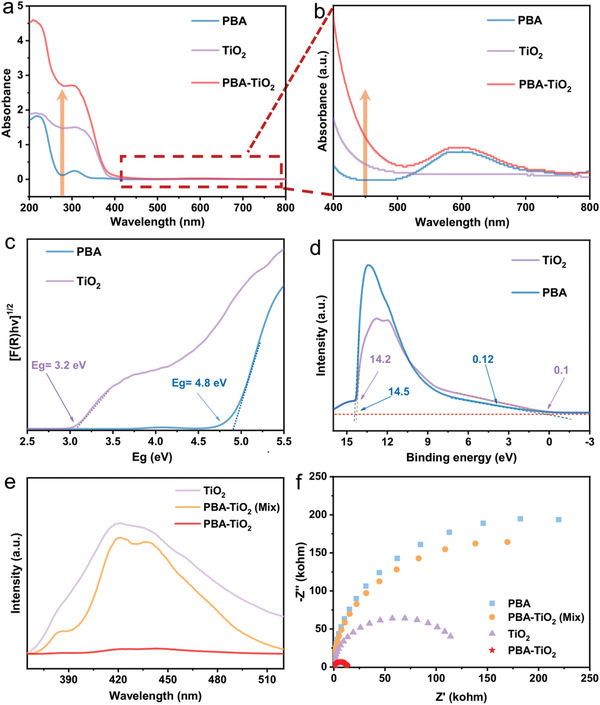
a) UV–vis absorption spectra. b) Partial graph of UV–vis absorption spectra. c) Tauc plot of sample TiO_2_ and PBA for determining the band gaps. d) UPS spectra of TiO_2_, PBA for determining the VB position. e) PL emission spectra (excitation wavelength: 340 nm). f) Nyquist plots of pristine TiO_2_, PBA, PBA–TiO_2_ Janus nanoreactors, and PBA–TiO_2_ (mix) under simulated solar illumination.

In addition, UV−vis diffuse reflection spectra imply that the light absorption of the PBA–TiO_2_ Janus nanoreactor is obviously enhanced regardless of the UV and visible‐light range, in comparison with PBA and TiO_2_ alone (**Figure** **5**a,b). This testifies the strong interaction was formed between PBA and TiO_2_, in consistent with the XPS result. The band gap in Figure 5c is calculated to be 3.2 eV for TiO_2_ and 4.8 eV for PBA from the Tauc plot, respectively. Moreover, the ultraviolet photoelectron spectroscopy (UPS) is performed to determine the VB position of TiO_2_ and PBA (Figure 5d). The VB position was calculated to be 7.1 and 6.82 eV by subtracting the width of the He I UPS spectra from the excitation energy (21.2 eV). The CB positions of TiO_2_ and PBA are thus determined to be 3.9 and 2.02 eV from the difference between *E*_g_ and *E*_VB_.^[^ ^30^ ^]^ The band structure of PBA–TiO_2_ Janus nanoreactor indicated that it has the ability to achieve both photocatalytic water reduction and oxidation.

The separation and recombination property of photoexcited carriers were studied by PL spectra under illumination at 340 nm. As shown in Figure 5e, it is noted that the PBA–TiO_2_ Janus nanoreactor reveals a much lower PL peak, indicating the effective suppression of the charge carriers recombination in PBA–TiO_2_ Janus nanoreactor. More interestingly, due to the intimate interface between the PBA and TiO_2_ particles in PBA–TiO_2_ Janus nanoreactor, photoexcited carriers are rapidly transferring, leading to promoted charge separation. Furthermore, the time‐resolved fluorescence spectra were recorded to determine the lifetime of photoexcited carriers (Figures S10 and S11, Supporting Information). As shown in Table S2 (Supporting Information), the fluorescence lifetime of the PBA–TiO_2_ particles (1.78 ns) is longer than that of TiO_2_ (1.69 ns) and PBA–TiO_2_ (mix) (1.59 ns), indicating better charge transfer ability in Janus structure. Overall, the decreased PL intensity and increased fluorescent lifetime on PBA–TiO_2_ nanoreactor imply the suppressed radiative recombination with the long‐lived photoinduced electrons due to the strong interaction between TiO_2_ and PBA particles.

To investigate the charge‐transfer kinetics of the prepared samples, EIS was carried out by recording the Nyquist plots under simulated solar light (Figure 5f). It is worth noting that the semicircle of the PBA–TiO_2_ Janus nanoreactor was smaller than either the PBA, TiO_2_, and PBA–TiO_2_ (mix), implying the lowest interfacial charge‐transfer resistance.^[^ ^31^ ^]^ Stated thus, the PBA–TiO_2_ Janus nanoreactor exhibits a fantastic superiority in charge separation and transfer, benefiting from the strong interfacial interaction.

The SPV spectra were performed to explore the charge transfer dynamics in the PBA–TiO_2_ Janus nanoreactor. As shown in Figure S12 in the Supporting Information, an obvious positive SPV response for bare TiO_2_, which implies the typical feature of an n‐type semiconductor. It is widely accepted that for a similar composition of semiconductors, the photovoltage intensity is positively correlated to the separation efficiency. Compared to bare TiO_2_ and PBA, the PBA–TiO_2_ Janus nanoreactor shows higher SPV value, confirming the efficient separation of photogenerated electron–hole pairs in the Janus structure.

**Figure 6:**
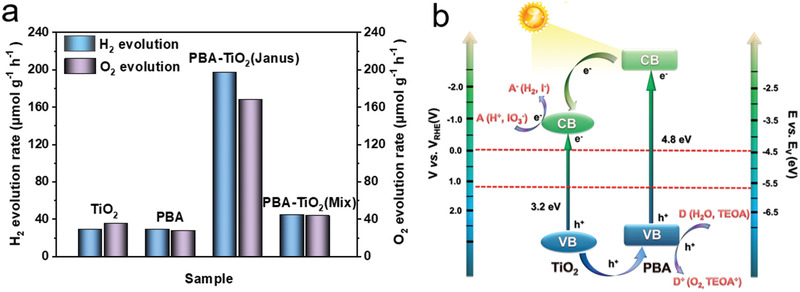
a) Comparison of photocatalytic H_2_ evolution and O_2_ evolution rate of TiO_2_, PBA, PBA–TiO_2_ Janus nanoreactors and PBA–TiO_2_ (mix) samples under light irradiation. b) Schematic representation of Janus nanoreactor for photocatalytic water splitting (A: electron acceptor; D: electron donor).

Enhanced by type II heterojunction structure, suitable band positions and superior charge separation ability, the PBA–TiO_2_ Janus nanoreactor is highly promising for solar water splitting. Accordingly, the photocatalytic performances of PBA–TiO_2_ Janus nanoreactor for water reduction and oxidation were examined by using TEOA and NaIO_3_ as hole and electron scavengers, respectively. As displayed in **Figure** **6**a, the PBA–TiO_2_ Janus nanoreactor exhibits the best performance with a H_2_ evolution rate of 198 µmol g^−1^ h^−1^ under light irradiation, which is almost 6.6 times higher than that of bare TiO_2_ and PBA, respectively. Similarly, the sample exhibits the optimal O_2_ evolution rate of 168 µmol g^−1^ h^−1^, which is about 5 times higher than that of bare TiO_2_ and PBA, respectively. It is worth noting that both photocatalytic H_2_ and O_2_ activity of PBA–TiO_2_ Janus nanoreactor is about fourfold that of the physical mixture of PBA and TiO_2_, indicating the unique advantage of the Janus structure. As listed in Tables S3 and S4 in the Supporting Information, the PBA–TiO_2_ Janus nanoreactor shows superior photocatalytic performance compared to many reported MOFs‐based materials.

PBA–TiO_2_ Janus nanoreactor also exhibits a H_2_ evolution rate of 78 µmol g^−1^ h^−1^ under visible light irradiation, higher than bare PBA (>420 nm) (Figure S13, Supporting Information). Besides, the amount of TiO_2_ can directly affect the photocatalytic performance (see Table S1 in the Supporting Information). The best performance was achieved for PBA–TiO_2_(c) as Janus nanoreactor. The highest H_2_ evolution rate reached 198 µmol g^−1^ h^−1^. In addition, the photocurrent–time curves were recorded through light turning up/off process of the chronoamperometry method in Figure S14 in the Supporting Information. The PBA–TiO_2_ Janus nanoreactor has the highest photocurrent density, which is 2.7, 4.0, and 8.0 times higher than those of pristine TiO_2_, PBA–TiO_2_ (mix) and PBA, respectively, which was consistent with the result of photocatalytic water splitting in Figure 6a.

A schematic diagram showing the separation and transport process of photoexcited electron–hole in the PBA–TiO_2_ Janus nanoreactor is illustrated in Figure 6b. The PL spectra, in combination with experimental characterization, lead to the conclusion that the enhanced photocatalytic activity of the Janus nanoreactor is mainly due to the suitable heterojunctions formed between the two materials (2D nanocrystal–single crystal) with different energy levels. Under light illumination, the photogenerated electrons were transferred from the VB to the CB for PBA and TiO_2_.^[^ ^32^ ^]^ Then, due to the heterojunction structure and the fact that the CB edge potential of PBA (2.02 eV) is more negative than that of TiO_2_ (3.9 eV), the photogenerated electrons in PBA could transfer to the CB of TiO_2_, while the holes in the VB of TiO_2_ could transfer to PBA. Most importantly, the interfacial structure constructed by this planting‐inspired strategy greatly promotes the electron transfer from PBA to TiO_2_, resulting in the promotion of the separation of photogenerated electron–hole pairs.

The stability of the photocatalysts is the key to the throughout photocatalytic water splitting process. Hence, we recovered the samples after the reaction. XRD and XPS techniques are employed to determine the stability of the recycled samples. As shown in Figure S16a in the Supporting Information, all the diffraction peaks of the recycled PBA–TiO_2_ Janus nanoreactor can be assigned to JCPDS card no. 89‐3738 without impurity peak. Simultaneously, the survey results of XPS on the recycled PBA–TiO_2_ Janus nanoreactors confirm that the compositions and chemical states are consistent with fresh PBA–TiO_2_ Janus nanoreactor (Figure S16a, Supporting Information). Moreover, the XRD and XPS results of the recycled samples including PBA–TiO_2_ (mix), PBA and TiO_2_ are similar with the fresh sample (Figures S15c,d and S16c,d, Supporting Information). These results indicate that the PBA–TiO_2_ Janus nanoreactors and two individual components are not changed after the photocatalytic reaction at local circumstance. The cycling tests of PBA–TiO_2_ Janus nanoreactors were performed to investigate the photocatalytic stability (Figure S17, Supporting Information). The rate of H_2_ evolution did not change significantly after three cycles reaction, indicating good stability of the PBA–TiO_2_ Janus nanoreactors under testing condition, which is consistent with the XRD and XPS results (Figure S18, Supporting Information).

## 3 Conclusion

In summary, we have demonstrated the anisotropic PBA–TiO_2_ Janus nanoreactor possessing superior photocatalytic water splitting performance, in which the unsaturated metal sites caused by PBA void surface are utilized as anchoring sites. The mild etching PBA crystals were used as the substrates for nucleation and growth of TiO_2_ 2D nanoflakes. The PBA–TiO_2_ Janus nanoreactor exhibited boosting photocatalytic activities for both water reduction and oxidation reactions compared with bare TiO_2_, PBA, and PBA–TiO_2_ (Mix) samples. This is attributed to the more efficient charge separation and transfer in Janus structures, as proved by UV–vis, XPS, EIS, PL, and SPV spectra. This planting‐inspired strategy reported here opens up a new window for the design and construction of Janus nanoreactor as efficient heterojunction photocatalysts.

## 4 Experimental Section

##### Synthesis of Ni–Co Prussian Blue Analog Cubes

In a typical synthesis, nickel nitrate (0.6 mmol) and sodium citrate (0.9 mmol) were dissolved in 20 mL of deionized (DI) water to form solution A. Potassium hexacyanocobaltate(III) (0.4 mmol) was dissolved in 20 mL of DI water to form solution B. Then, solutions A and B were mixed under magnetic stirring for 5 min and aged for 24 h at room temperature. The precipitate was collected by centrifugation, washed with water and ethanol, and dried at 70 °C overnight. For comparison, the controlled PBA cubes with an aging time of one day were also synthesized.

##### Synthesis of PBA–TiO_2_

20 mg of PBA cubes were dispersed into a mixed solution of acetic acid (5 mL) and glycol (40 mL) by ultrasonication, followed by addition of 0.4 mmoL of TiF_4_. After thorough mixing, the suspension was transferred into a 60 mL Teflon‐lined stainless steel autoclave and heated at 180 °C for 8 h. After the autoclave was cooled down to room temperature, the resultant product was centrifuged and washed with deionized water and ethanol, and dried at 70 °C overnight. The different proportions of PBA and TiO_2_ composites were prepared by changing the amount of TiF_4_, and labeled as a) PBA–TiO_2_, b) PBA–TiO_2_, c) PBA–TiO_2_, d) PBA–TiO_2_, and e) PBA–TiO_2_.

##### Photocatalytic H_2_/O_2_ Evolution Measurements

Photocatalytic H_2_/O_2_ evolution reactions were carried out in a top‐irradiation vessel connected to a glass closed gas circulation system. 10 mg of the photocatalyst powder was dispersed in 100 mL aqueous solution containing 5 vol% triethanolamine/0.01 m NaIO_3_ as hole/electron scavenger. The amount of evolved H_2_/O_2_ was determined using a gas chromatography (Agilent 6890). The light source was a 300 W Xe lamp.

## Conflict of Interest

The authors declare no conflict of interest.

## Supporting information

Supporting Information
