# Supplementary material for: Modular Construction of Prussian Blue Analog and TiO2 Dual‐Compartment Janus Nanoreactor for Efficient Photocatalytic Water Splitting
Source: Adv Sci (Weinh). 2021 Feb 16;8(7):2001987. doi: 10.1002/advs.202001987 (PMC8024990; doi:10.1002/advs.202001987)
Supplement: Supplementary file 1 — Supporting Information [file ADVS-8-2001987-s001.pdf]

© 2021 Wiley-VCH GmbH

ADVANCED SCIENCE
Open Access

Supporting Information

for *Adv. Sci.,* DOI: 10.1002/advs.202001987

Modular Construction of Prussian Blue Analogue and TiO_2 Dual-Compartment Janus Nanoreactor for Efficient Photocatalytic Water Splitting

Chunjing Shi,[#] Sheng Ye,[#] Xuewen Wang, Fanning Meng, Junxue Liu, Ting Yang, Wei Zhang, Jiatong Wei, Na Ta, Gao Qing (Max) Lu, Ming Hu, and Jian Liu**

Supporting Information

Modular Construction of Prussian Blue Analogue and TiO_2 Dual-Compartment Janus Nanoreactor for Efficient Photocatalytic Water Splitting

Chunjing Shi,[#] Sheng Ye,[#] Xuewen Wang, Fanning Meng, Junxue Liu, Ting Yang, Wei Zhang, Jiatong Wei, Na Ta, Gao Qing (Max) Lu, Ming Hu,[*] and Jian Liu[*]

[#] These authors contributed equally to this work and should be regarded as co-first authors.

1. Materials and Characterization:

Chemicals:

All the chemicals were analytical grade and used without further purification. The nickel nitrate, sodium citrate, potassium hexacyanocobaltate and titanium tetrafluoride were obtained by Aladdin (Shanghai) Technology Co.,Ltd. Deionized water (R = 18.25 MΩ) was used in the whole experiment.

Characterization

The crystal structure of the resultant products was characterized by X-ray diffraction (XRD) using a Rigaku Smartlab with Cu Kα radiation (λ = 1.5418 Å) over the 2θ range of 10°-80° with a scan speed of 5°/min at room temperature. The scanning electron microscopy (SEM) were performed with a JEOL JSM-6360 scanning electron microscope operating at an acceleration voltage of 20 kV. The high-resolution TEM and energy dispersive X-ray (EDX) spectroscopy were carried out on a JEM-2100F with an acceleration voltage of 120 kV. X-ray photoelectron spectroscopy (XPS) was conducted on a thermofisher ESCALAB 250Xi spectrometer using a monochromated Al Kα excitation source (hυ=1486.6eV). All energies were calibrated to spurious carbon at 284.6 eV. Ultraviolet photoelectron spectroscopy (UPS) measurements were performed with an unfiltered HeI (21.2 eV) gas discharge lamp and a total instrumental energy resolution of 100 meV. The Fourier transform infrared (FTIR) spectrum is recorded on a FTIR spectrometer (Spectrum One, Perkin Elmer) using a standard KBr pellet technique. Raman measurements were performed by Renishow spectrometer using a laser with a excitation wavelength of 532 nm. Room-temperature UV−vis diffuse reflectance spectroscopy (DRS) were performed between 800 and 200 nm on a Shimadzu UV-2600 instrument. Decay curves of the as-made photocatalysts were obtained on a FLS920 fluorescence lifetime spectrophotometer (Edinburgh Instruments, UK) under the excitation of a hydrogen flash lamp with the wavelength at 340 nm (nF900; Edinburgh Instruments). The

digested samples were analyzed using the Inductively Coupled Plasma Optical Emission Spectrometer (ICPOES) (instrument model: Aglient 5110). The surface photovoltage measurements (SPV) were conducted by an instrument assembled with a photovoltaic cell where the photocatalyst powders are caught between two ITO electrodes (d = 1.2 mm, resistivity: 8–12 Ω cm^{-2}, Sigma-Aldrich, USA). A lock-in amplifier (SR830 DSP, Stanford Research Systems, USA) was applied to amplify the photovoltage signals of samples and a 500 W xenon lamp (CHF-XM-500W, Beijing Perfect Light Co., China) was used as the optical source, coupled with a grating monochromator (Omni-λ3007, Zolix, China) to provide monochromatic light. No external bias was applied.

Photoelectrochemical (PEC) property measurements:

Electrochemical impedance spectroscopy (EIS) and the photocurrent-time curves of the samples was evaluated in 1 M Na_2SO_4 electrolyte in a three-electrode quartz cell, and were performed on an electrochemical workstation (CHI660d, CH instruments, Inc.). AM 1.5 G (100 mW cm^{-2}) sunlight simulation was used as the light source. A platinum electrode was used as the counter electrode, and a saturated calomel electrode (SCE) electrode selected as the reference electrode. PBA-TiO_2 Janus nanoreactor, PBA, TiO_2 and PBA-TiO_2 (Mix) were prepared as working electrodes. Photocatalytic H_2-evolution rate is calculated during the reaction time of 0.5 h.

2. Supporting Figures:

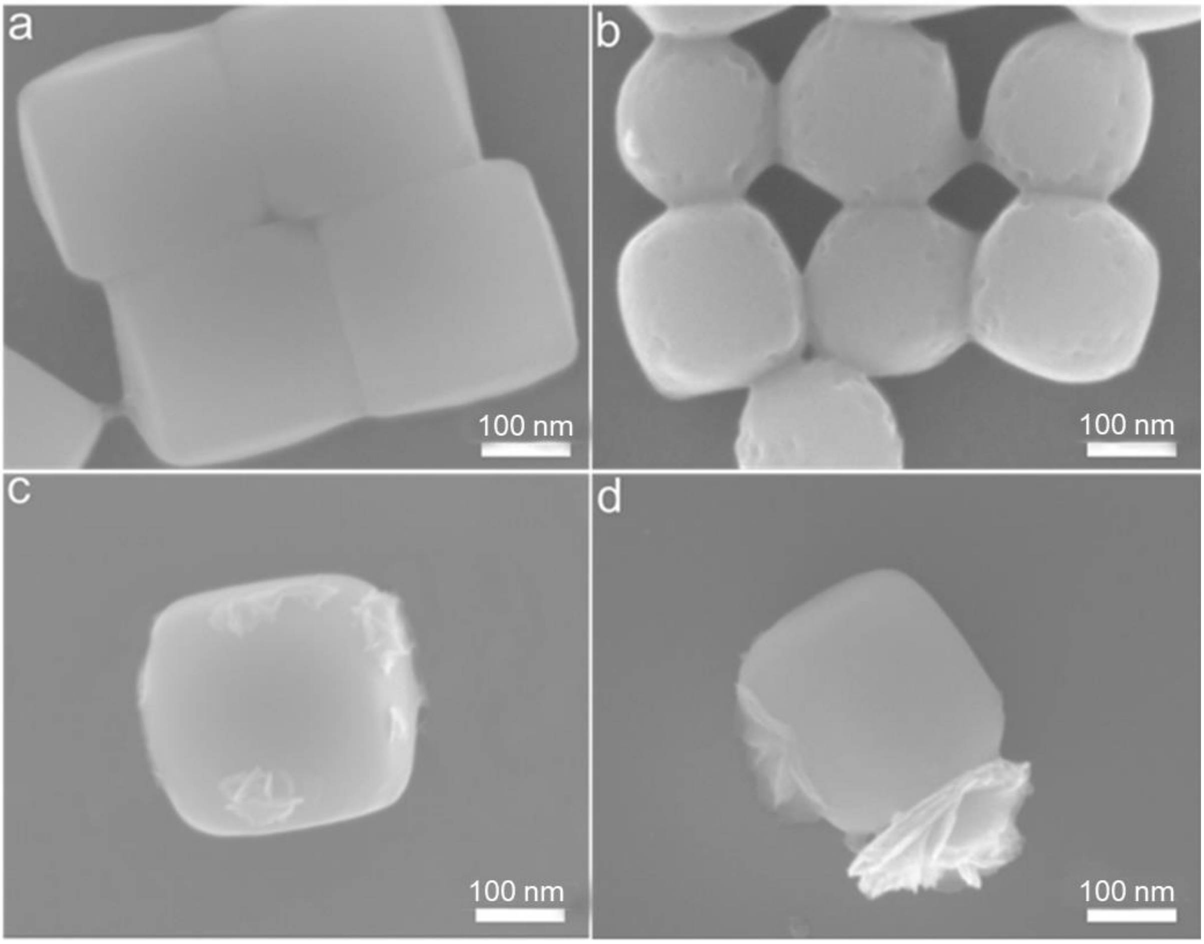

Figure S1. SEM images of (a) bare PBA, (b) etched PBA, (c) TiO_x species rooted into the PBA and (d) the resultant PBA-TiO_2 Janus nanoreactors.

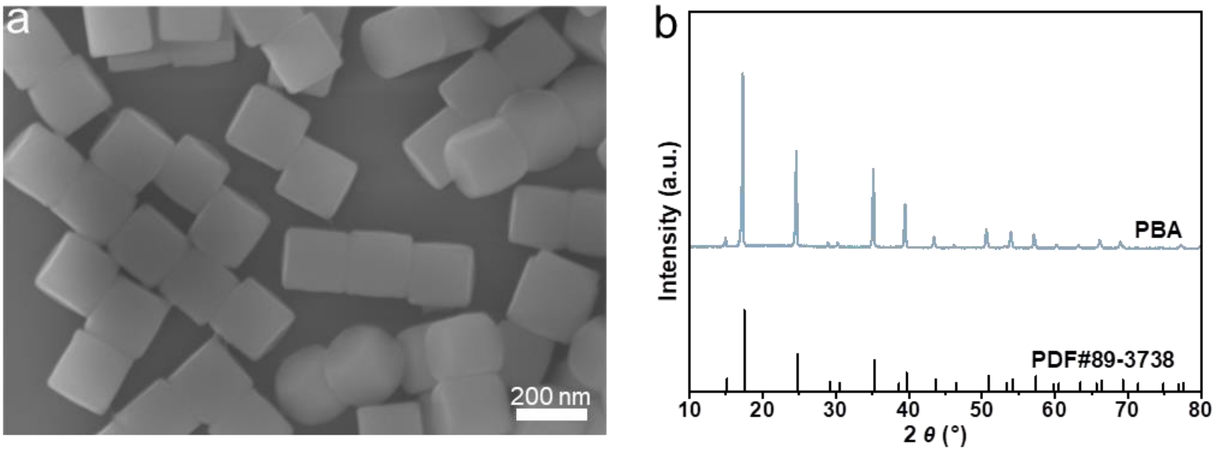

Figure S2. (a) SEM image and (b) XRD pattern of the as-prepared PBA sample.

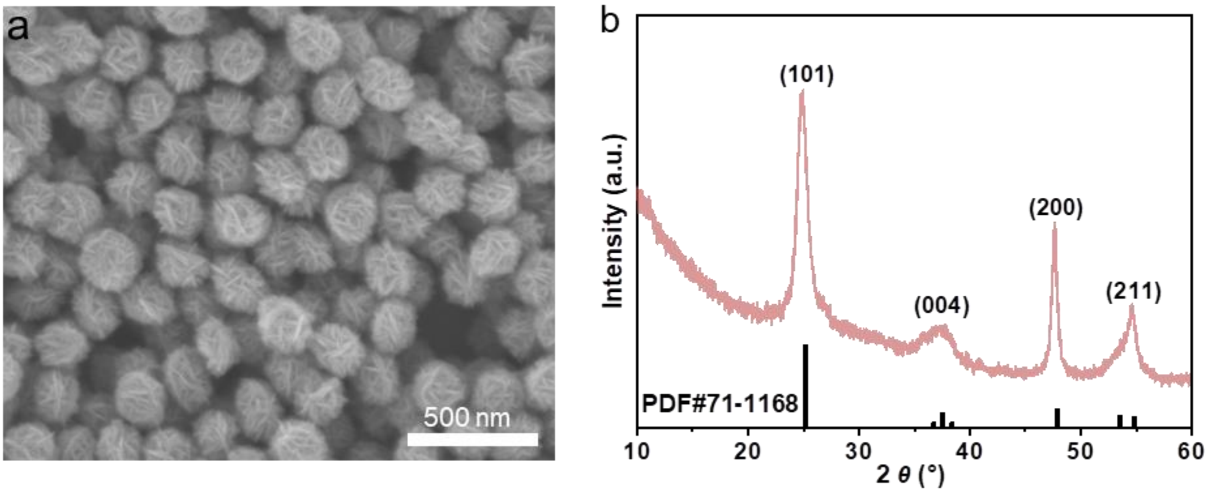

Figure S3. (a) SEM image and (b) XRD pattern of the as-prepared TiO_2 sample.

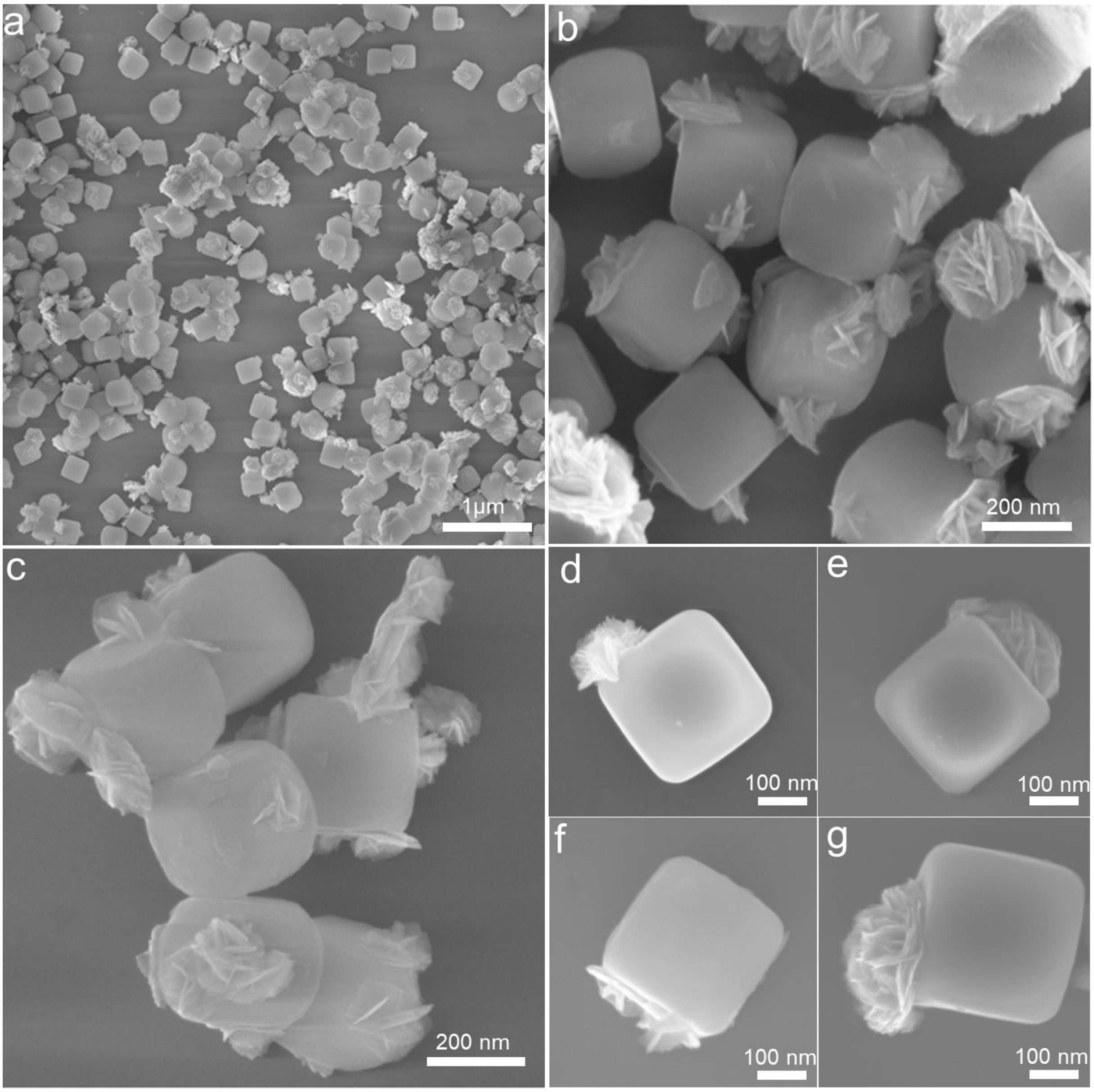

Figure S4. (a-g) SEM images of the as-prepared PBA-TiO_2 Janus nanoreactors. The PBA-TiO_2 Janus structure is uniform, where the flower-like TiO_2 is grown on the surface of PBA cubes.

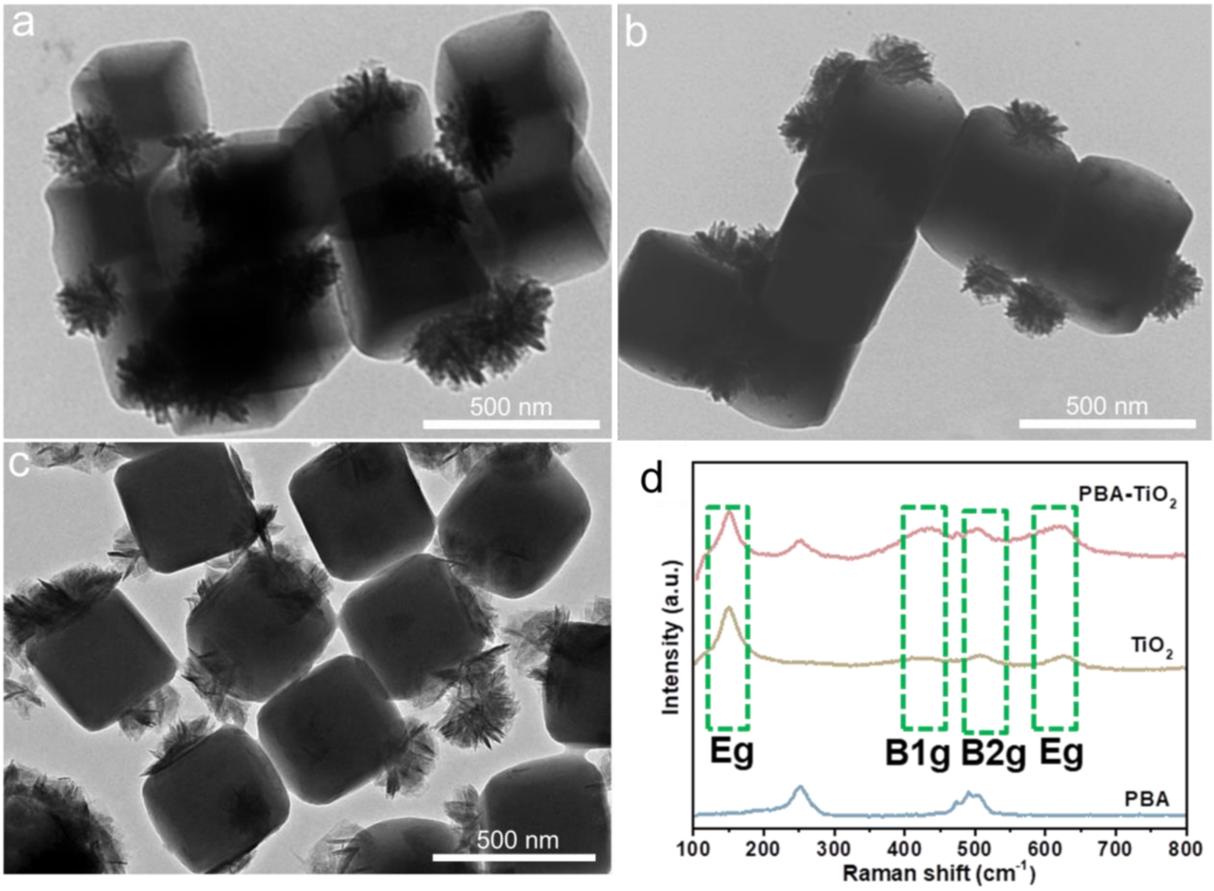

Figure S5. (a-c) TEM images of the as-prepared PBA-TiO_2 Janus nanoreactors at various resolutions. The PBA-TiO_2 Janus nanoreactors contains a PBA cube and the flower-like TiO_2 with the nanoflakes. (d) Raman profiles of the as-prepared PBA, TiO_2 and PBA-TiO_2 Janus particles.

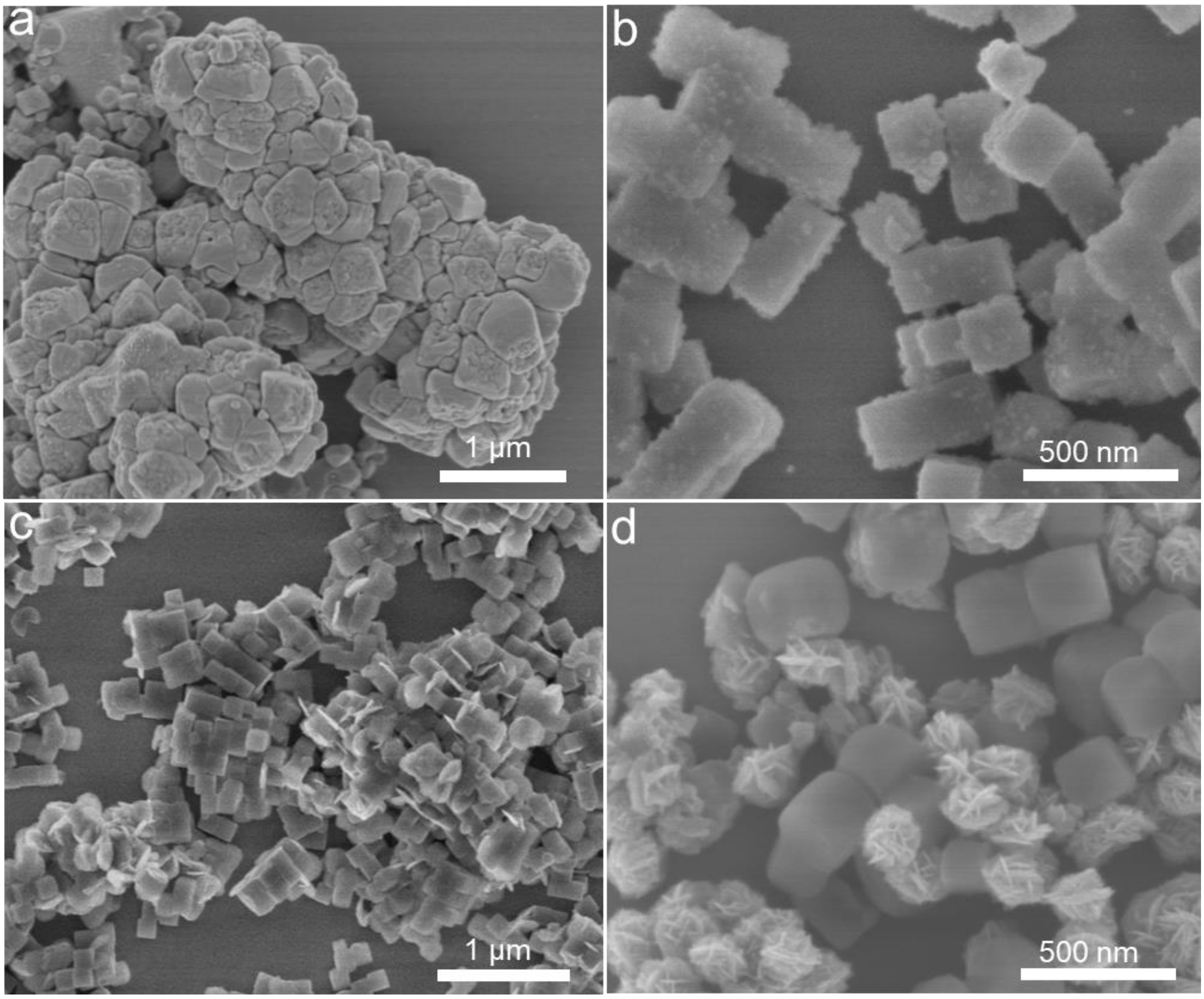

Figure S6. SEM images of the PBA-TiO_2 prepared (a) in the case of glycol and hydrochloric acid solution at 180 ^{o}C for 8 h, (b) in the case of deionized water at 180 ^{o}C for 8 h and (c) in the case of acetic acid and glycol at 160 ^{o}C for 8 h, (d) SEM image of the physical mixture of PBA and TiO_2.

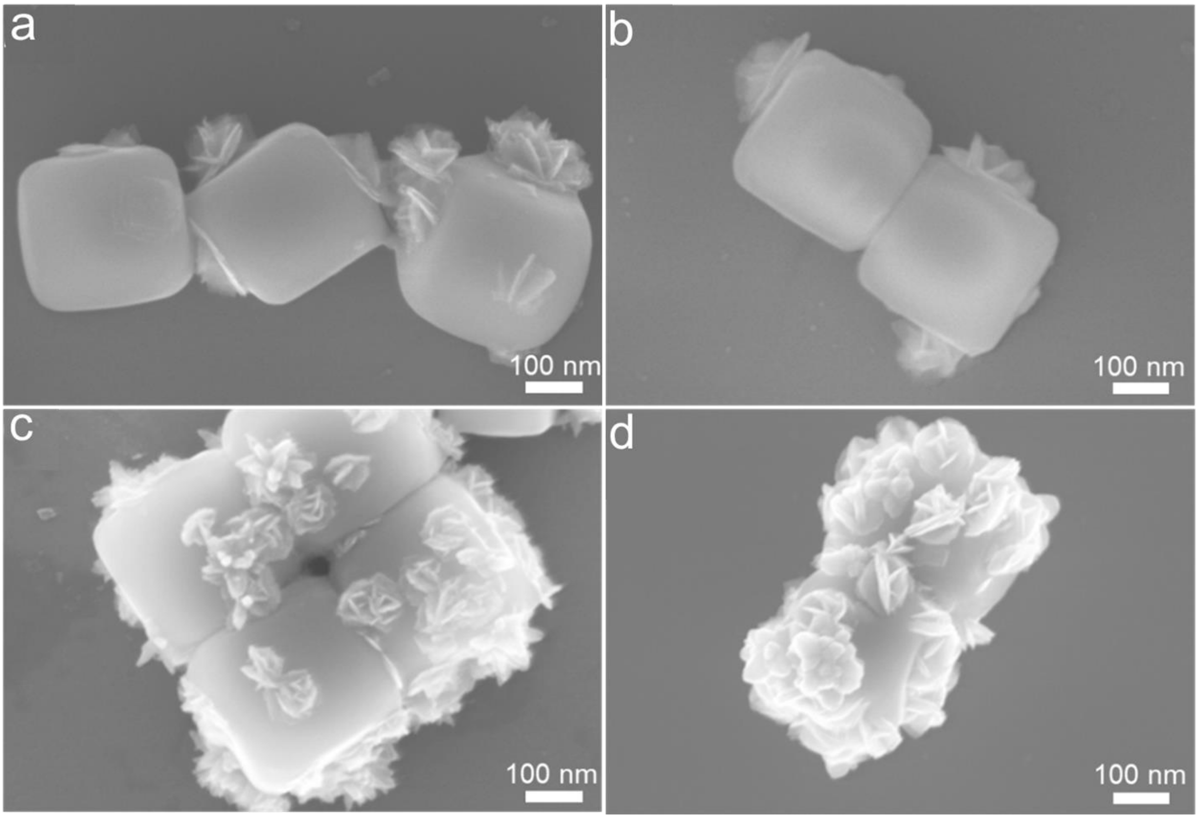

Figure S7. SEM images of the obtained PBA-TiO_2 samples from different contents of TiF_4 with (a, b) 0.4 mmol and (c, d) 1.3 mmol.

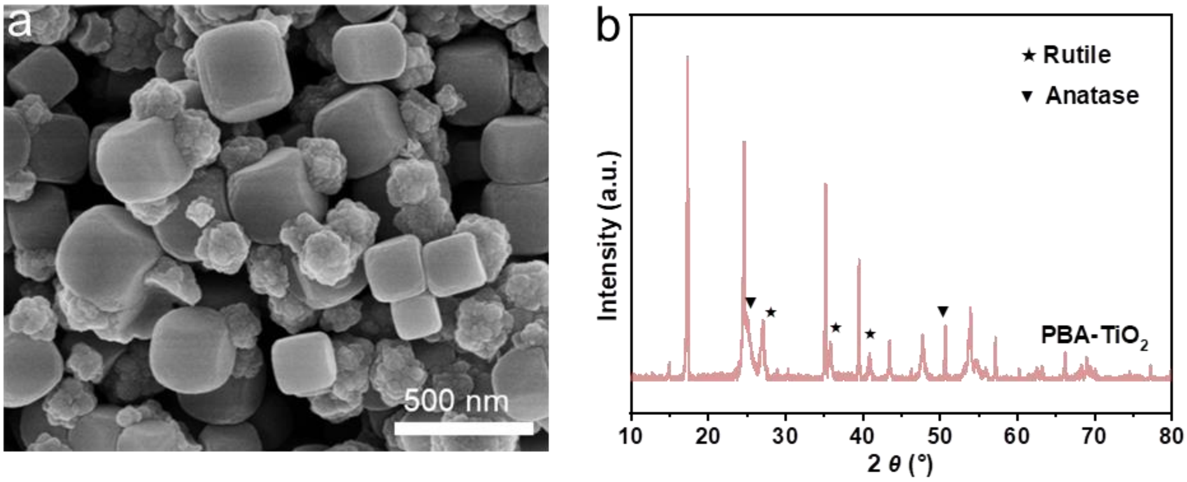

Figure S8. (a) SEM image and (b) the corresponding XRD pattern of the PBA-TiO_2 sample prepared by utilizing PBA without moisture removal.

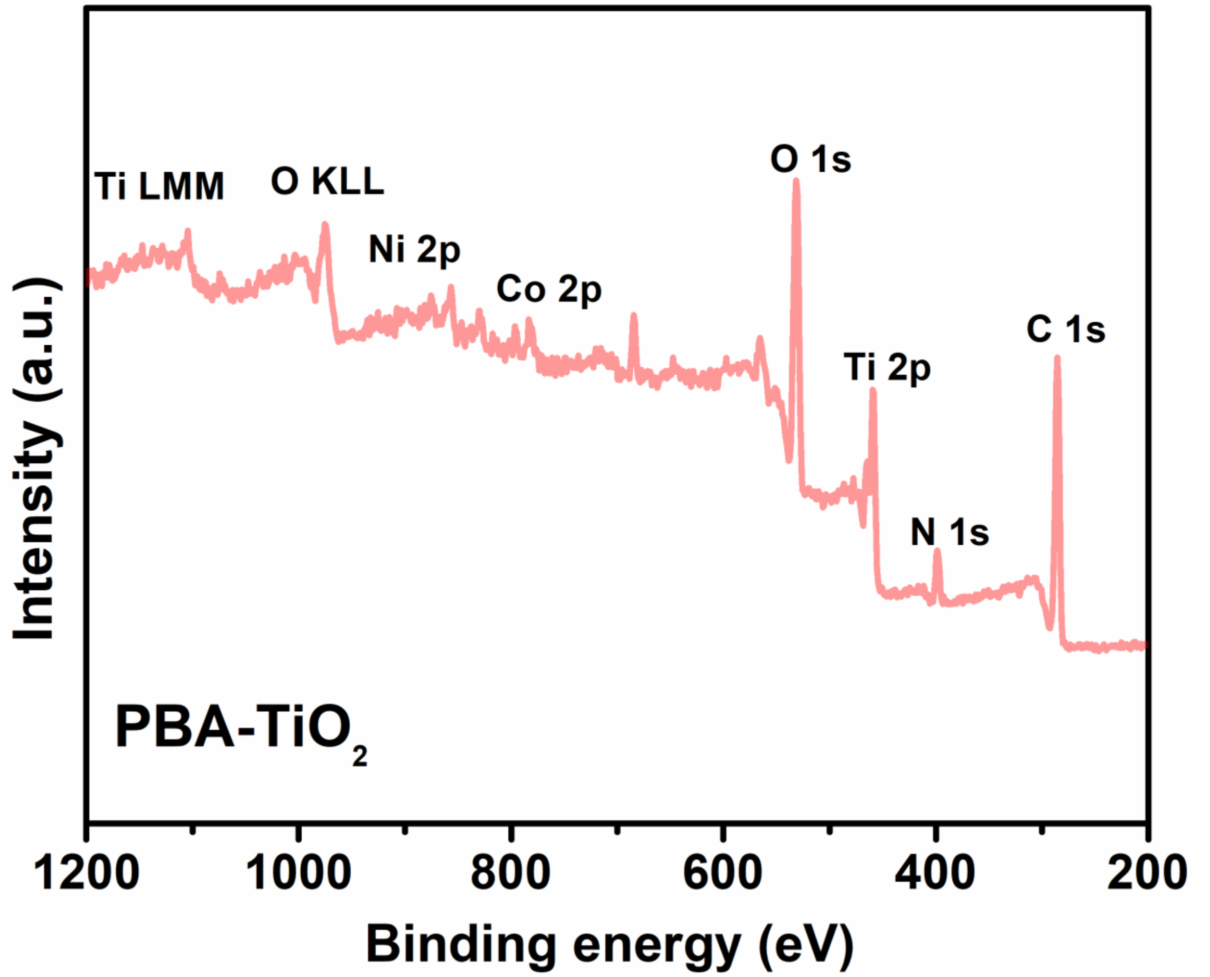

Figure S9. Survey XPS scan of PBA-TiO_2 Janus nanoreactor.

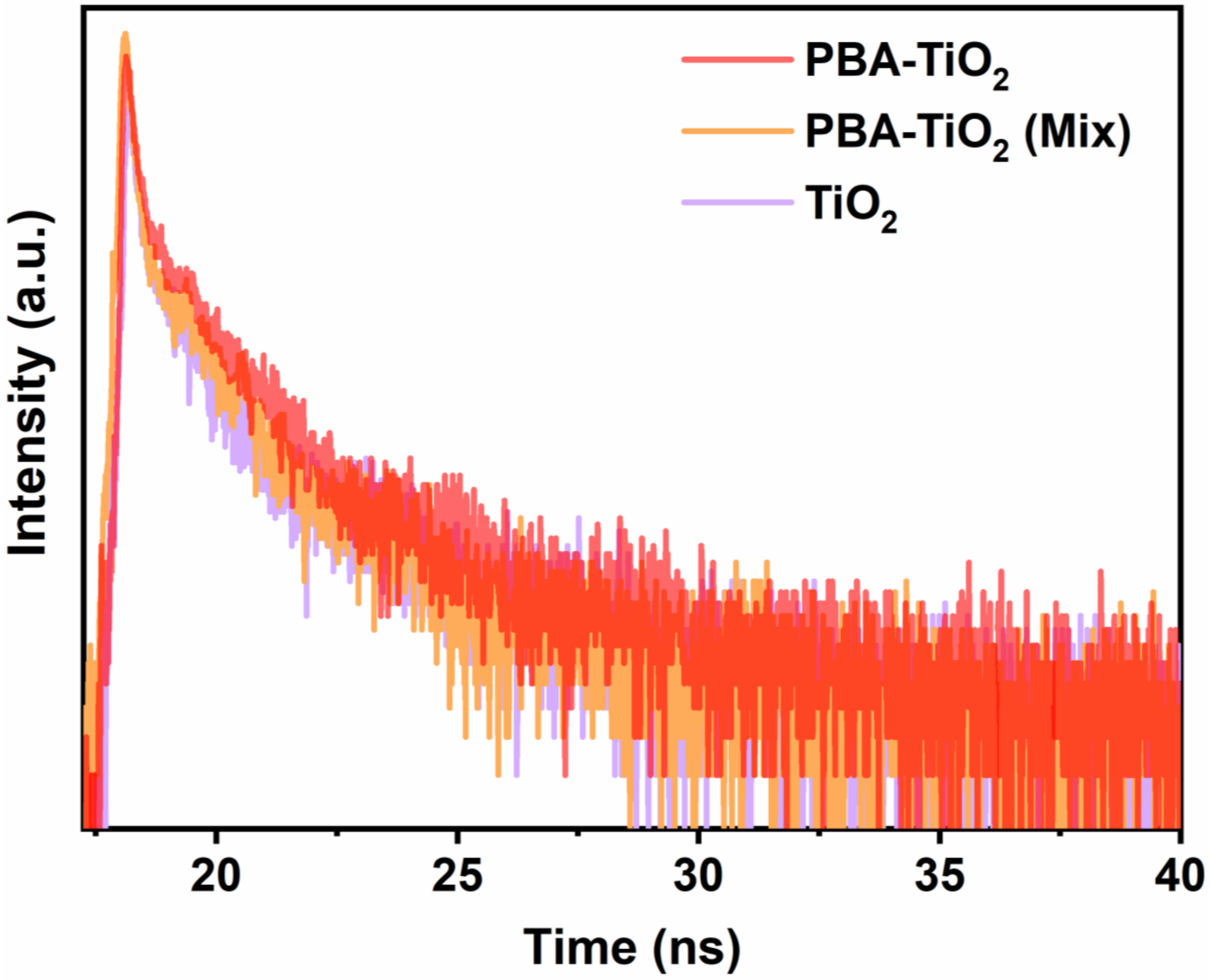

Figure S10. time-resolved transient PL decay of the obtained samples.

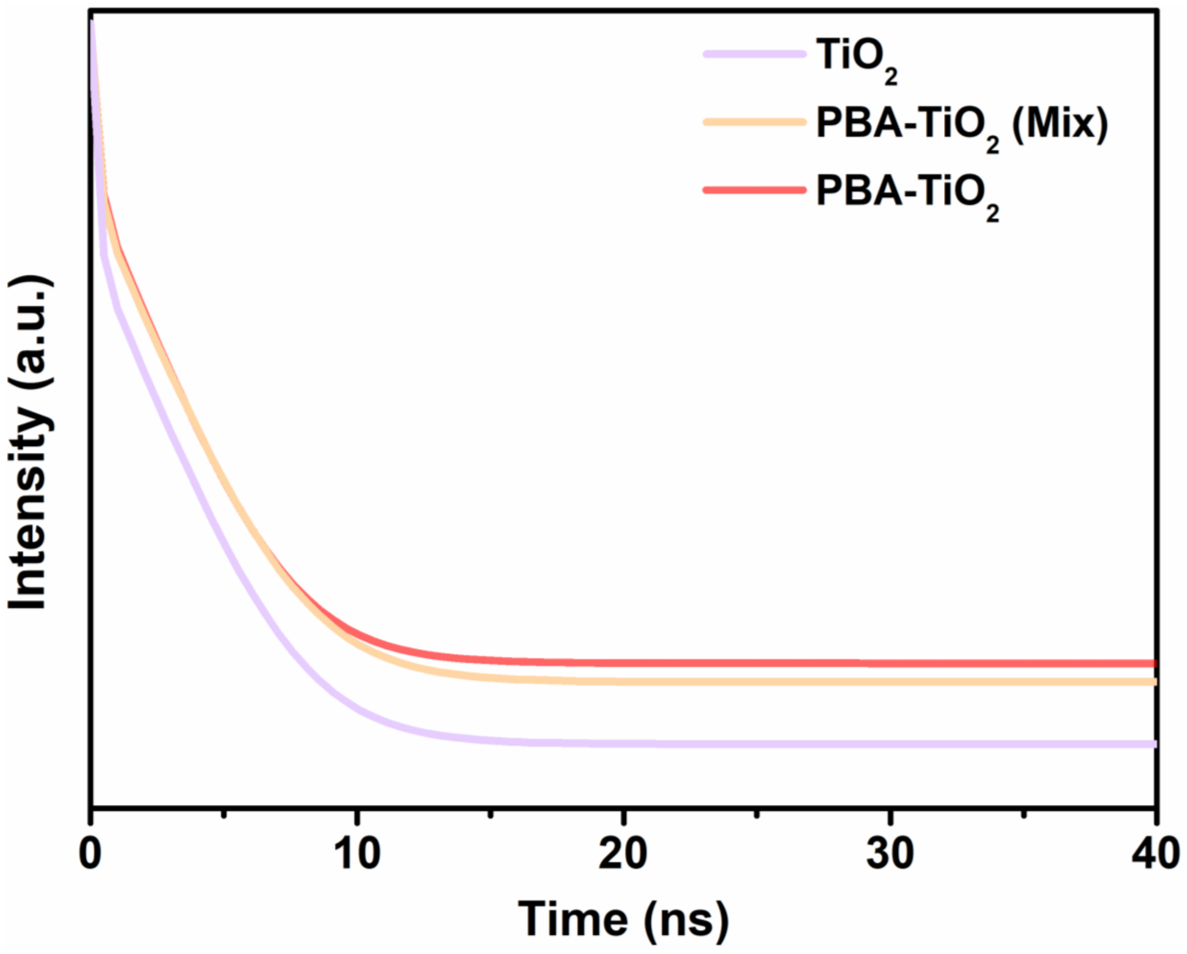

Figure S11. Fitted data of time-resolved transient PL decay of the as-prepared TiO_2, PBA-TiO_2 (Mix) and PBA-TiO_2 Janus nanoreactors. The calculated average PL lifetime (Table S1, Supporting Information) of the PBA-TiO_2 Janus nanoreactors is 1.78 ns, which is higher than that of PBA-TiO_2 (Mix) samples, indicating that a better interfacial charge transfer in the Janus structure.

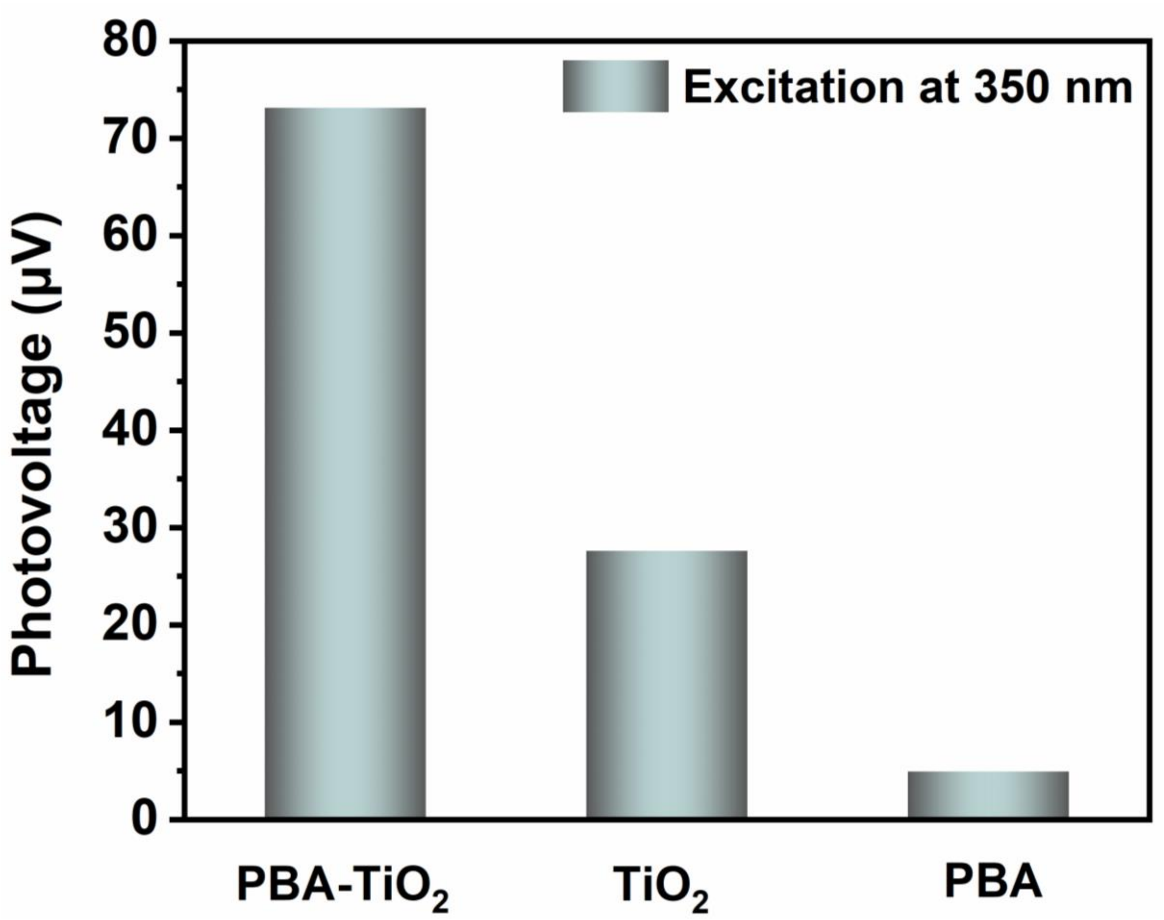

Figure S12. Comparison of the SPV spectra for bare PBA, TiO_2 and PBA-TiO_2 nanoreactor (the surface photovoltage signal in excitation wavelength 350 nm).

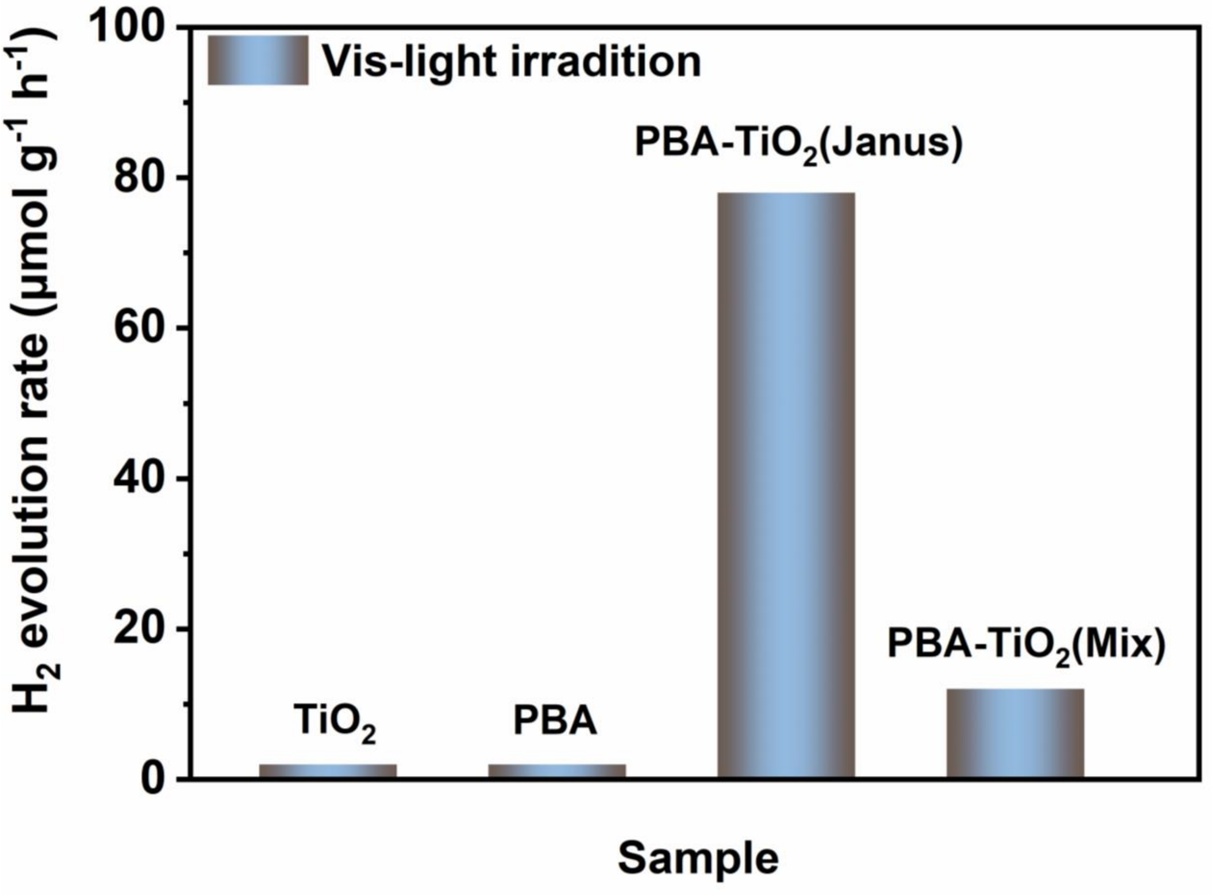

Figure S13. Comparison of photocatalytic H_2 evolution rate of TiO_2, PBA, PBA-TiO_2 Janus nanoreactors and PBA-TiO_2 (Mix) samples under vis-light irradiation.

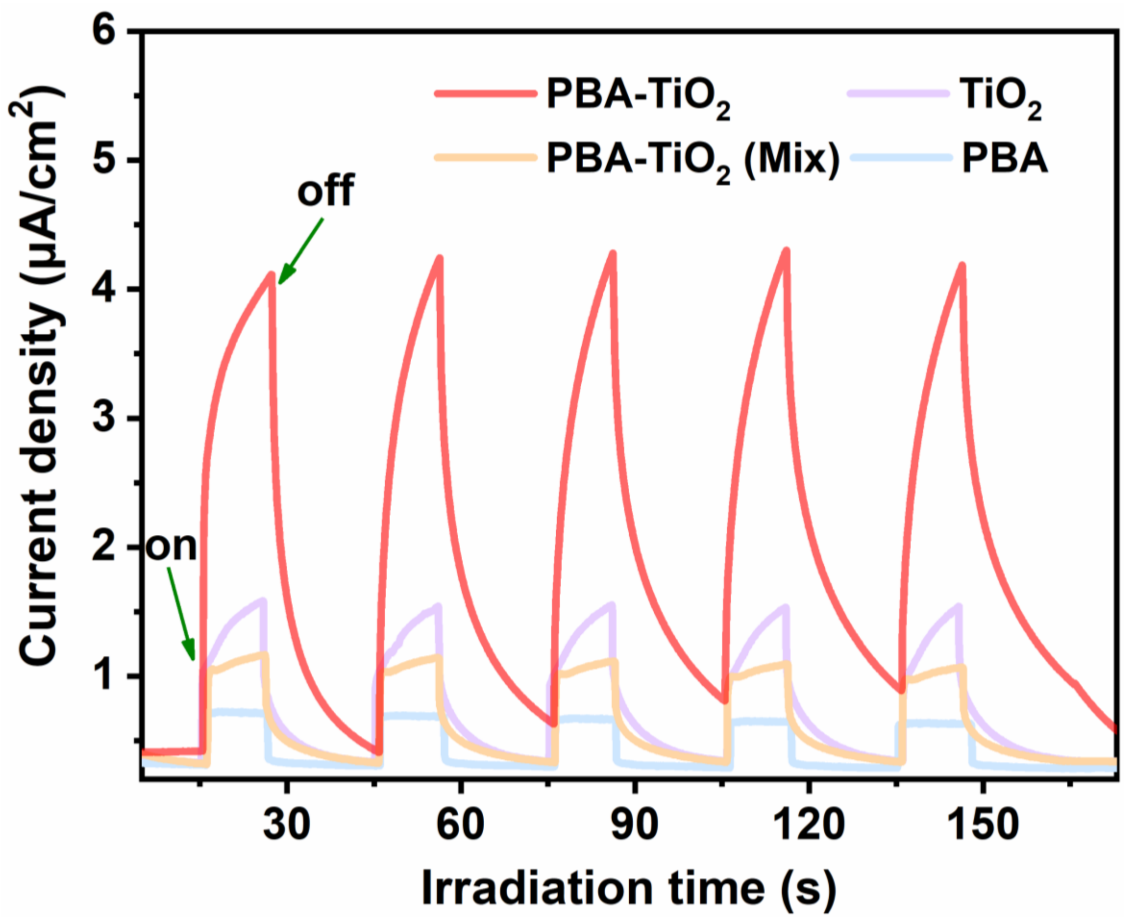

Figure S14. Photocurrent–time (I–t) curves measured with several typical on–off cycles of PBA, TiO_2, PBA-TiO_2 (Mix) and PBA-TiO_2 Janus nanoreactors photoelectrodes at 1.23 V vs. RHE in 1.0 M Na_2SO_4. The PBA-TiO_2 Janus particles exhibit higher photocurrent density than TiO_2, PBA and PBA-TiO_2 (Mix), respectively, in accordance with the action of photocatalytic water splitting.

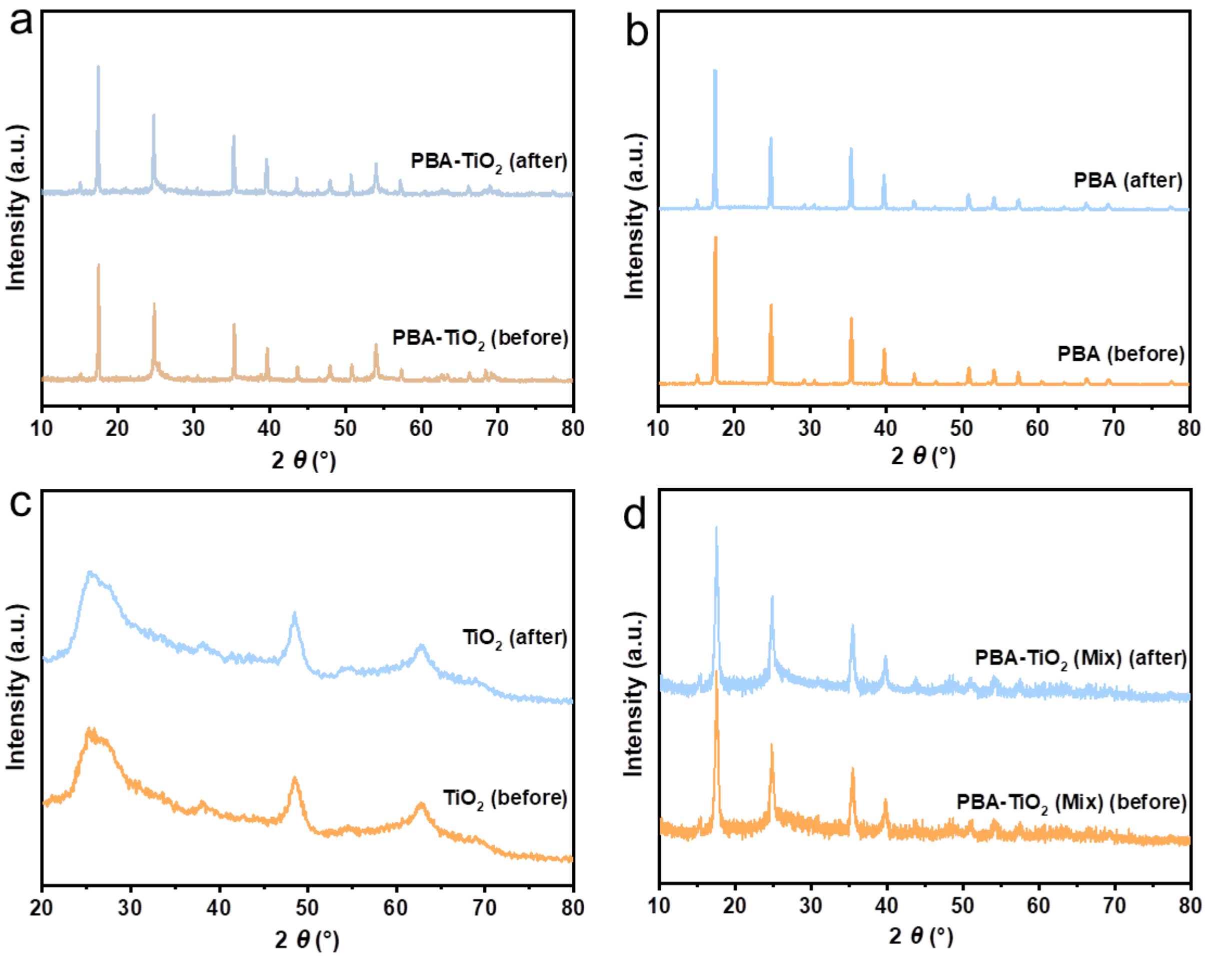

Figure S15. XRD patterns of (a) PBA-TiO_2 Janus nanoreactors, (b) PBA, (c) TiO_2 and (d) PBA-TiO_2 (Mix) before and after the reaction of photocatalytic water splitting. Note that all the prepared samples used for photocatalytic water splitting maintain the crystalline phase of the materials themselves.

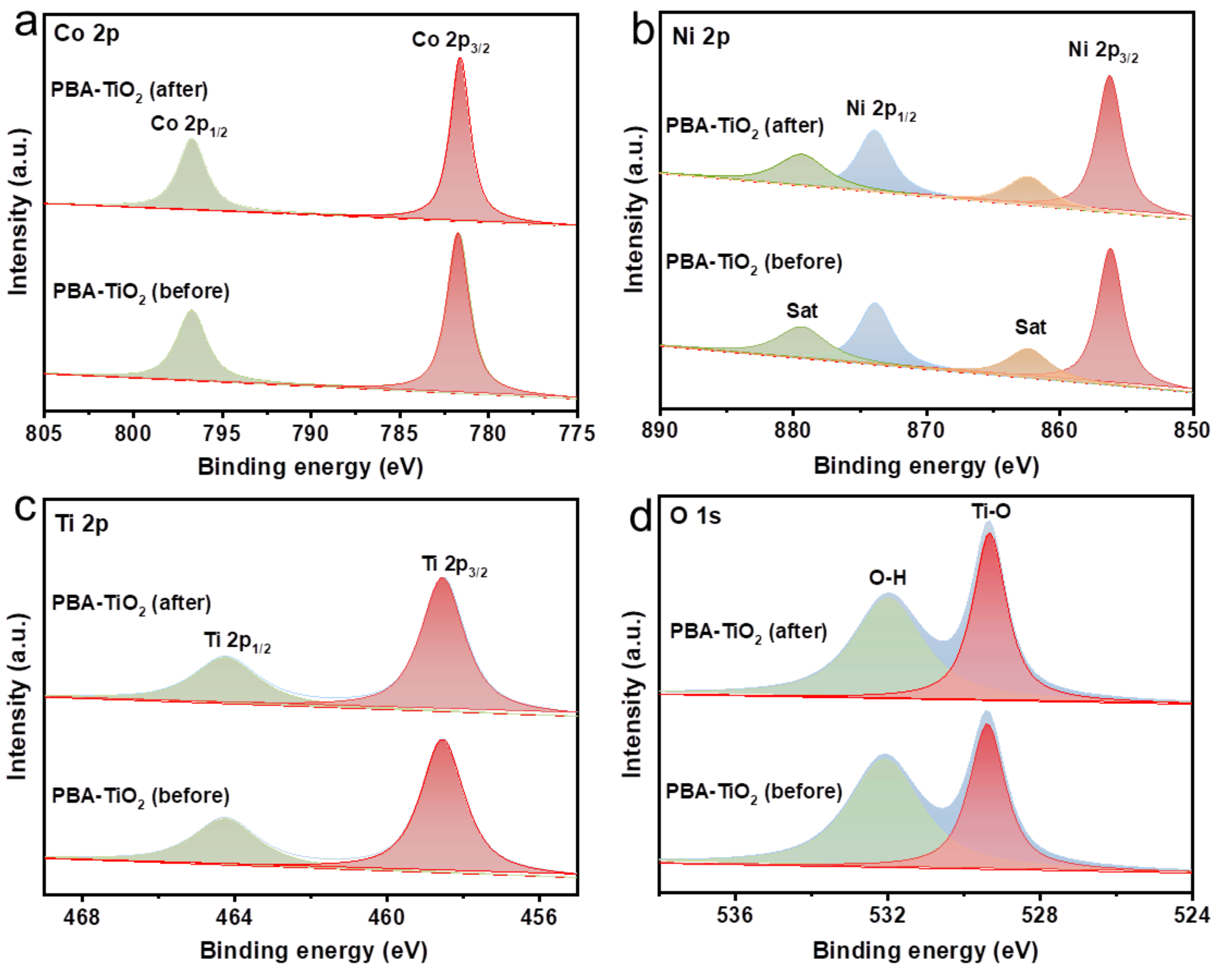

Figure S16. XPS (a) Co 2p analysis, (b) Ni 2p analysis, (c) Ti 2p analysis, and (d) O 1s spectra of the PBA-TiO_2 (before) and the PBA-TiO_2 (after) used for photocatalytic water splitting, revealing that the element Ni, Co, Ti and O peak positions have not been shifted obviously.

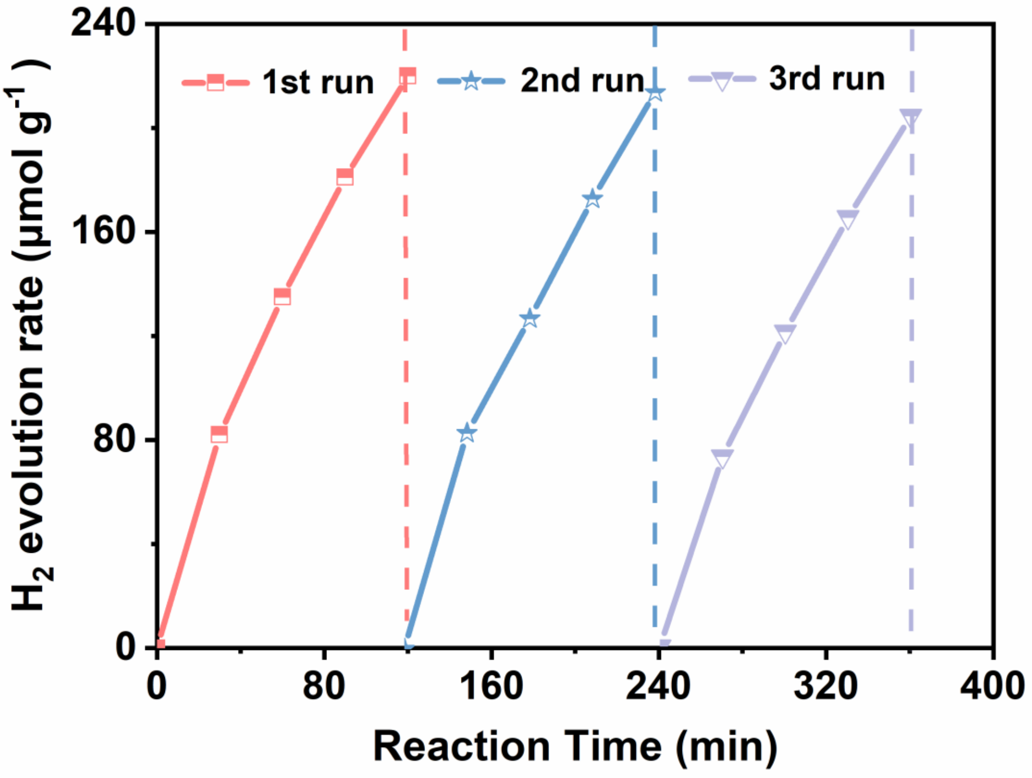

Figure S17. Cycling tests of photocatalytic hydrogen evolution for PBA-TiO_2 Janus nanoreactors.

The stability of the PBA-TiO_2 Janus nanoreactor was first investigated by repetitive water reduction experiments. As shown in Figure S18, no significant change in the total amount of H_2 evolution was observed for the three recycling runs. These results indicate that PBA-TiO_2 Janus nanoreactors have excellent activity and stability.

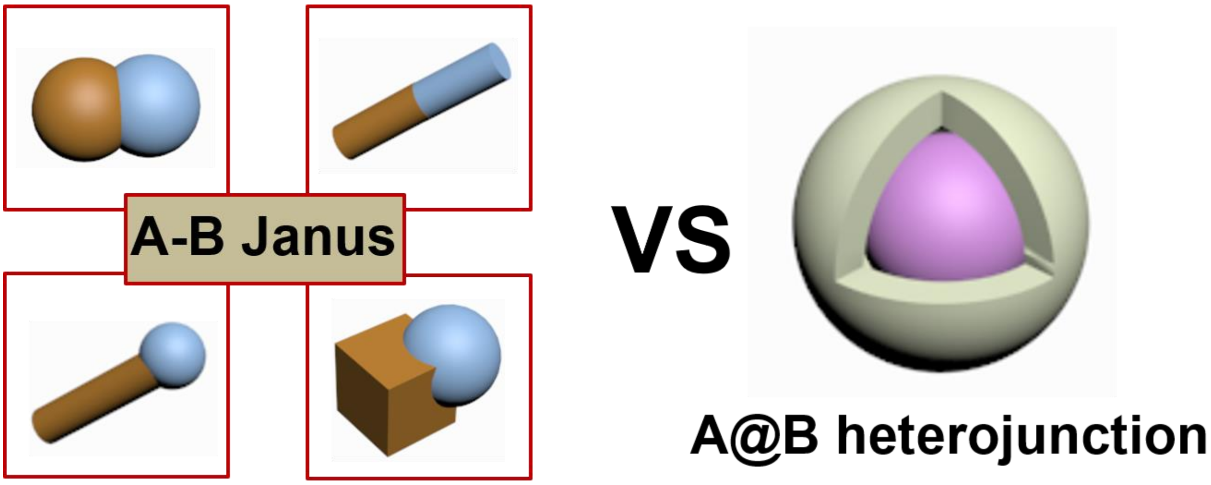

Figure S18. Schematic illustration of typical A-B Janus nanoarchitectures and A@B heterojunction.

As shown in Figure S18, the two compartment of A-B Janus nanoarchitectures both stands alone and exist interfacial region as a bridge for charge transfer. Therefore, the independent components can take advantage of the material itself and cooperate with each other through the interface. In contrary, for A@B heterojunction, the materials as core are wrapped up, it is difficult to display its unique advantages.

Table S1. The mass percentage of Ti in the composite was determined by the ICP measurements and the photocatalytic H_2 production activity.

Samples	Element mass ratio (Ni:Ti)	Element mass ratio (Co:Ti)	H_2 evolution rate (μmol g^{-1} h^{-1})
PBA-TiO_2(a)	0.3652	0.5448	102
PBA-TiO_2(b)	0.2683	0.1962	146
PBA-TiO_2(c)	0.2369	0.1417	198
PBA-TiO_2(d)	0.1582	0.1189	160
PBA-TiO_2(e)	0.1527	0.1159	108

The effective amount of TiO_2 (see Table S1) was evaluated by Inductively Coupled Plasma (ICP) analysis. All the catalysts were prepared twice in order to check for reproducibility. In all the cases, the measured values have been found to be within the error range. These results provide strong evidence for a large reliability of the method we used for the preparation of catalysts. The comparison of photocatalytic H_2 production activity of different weight percentage of TiO_2 in the composite under light irradiation.

Table S2. Summary of the photoluminescence decay time (τ) and average lifetime.

Sample	τ_1 (ns)	τ_2 (ns)	Avlifetime (ns)
TiO_2	2.02	0.18	1.69
PBA-TiO_2 (Janus)	0.17	2.12	1.78
PBA-TiO_2 (Mix)	0.14	2.04	1.59

Table S3. Comparison of photocatalytic H_2-evolution activities of MOFs-based composites.

Photocatalyst	Light source (sacrificial agent)	Conversion (μmol g^{-1} h^{-1})	Reference
PBA-TiO_2 (Janus)	300W Xe lamp (TEOA)	198	This work
Pt/$[Cu(en)_2]_4[PNb_{12}O_{40}(VO)_6]\cdot(OH)_5\cdot 8H_2O$	125 W Hg lamp (aq.CH_3OH)	44.35	[1]
RhB/UiO-66(Zr)-100*	300 W Xe lamp (TEOA)	100	[2]
Ti-MOF-$Ru(tpy)_2$	500 W Xe lamp (TEOA)	35	[3]
Pt-UiO-66-30	300 W xenon lamp (CH_3OH)	153	[4]
NH_2-MIL-125(Ti)	500 W Xe lamp (TEOA)	160	[5]
Pt/MIL-125(Ti)	Xe lamp (λ = 320–780 nm) (TEOA)	154	[6]
MIL-125-NH_2 (Pt)	Xe lamp (TEOA)	336	[7]
UiO-66-[FeFe](dcbdt)$(CO)_6$	300W Xe lamp	233	[8]
UiO-66/CdS/RGO	300W Xe lamp	210	[9]
Al-PMOF (MOF)	300W Xe lamp (EDTA)	200	[10]
Al(OH)(bpydc)·0.5$PtCl_2$	300 W Xe lamp (EDTA)	100	[11]
Calix-3/Pt@UiO-66-NH_2-1-200	300W Xe lamp	152.8	[12]

Table S4. Comparison of photocatalytic O_2-evolution activities of MOFs-based composites.

Photocatalyst	Light source (sacrificial agent)	Conversion (μmol g^{-1} h^{-1})	Reference
PBA-TiO_2 (Janus)	300 W Xe lamp ($NaIO_3$)	168	This work
MIL-88(Fe)	500 W Xe lamp ($AgNO_3$)	56	[13]
MIL-88-4H	500 W Xe lamp ($AgNO_3$)	52	[14]
MIL-53(Fe)	500 W Xe lamp ($AgNO_3$)	185	[15]
Bi-mna	500 W Xe lamp (AgNO3)	480	[16]

Supplementary references:

[1] J.-Q. Shen, Y. Zhang, Z.-M. Zhang, Y.-G. Li, Y.-Q. Gao, E.-B.Wang, *Chem. Commun.* **2014**, *50*, 6017.

[2] J. He, J. Wang, Y. Chen, J. Zhang, D. Duan, Y. Wang, Z. Yan, *Chem. Commun*. **2014**, *50*, 7063.

[3] T. Toyao, M. Saito, S. Dohshi, K. Mochizuki, M. Iwata, H. Higashimura, Y. Horiuchi, M. Matsuoka, *Chem. Commun*. **2014**, *50*, 6779.

[4] Y.-P. Yuan, L.-S. Yin, S.-W. Cao, G.-S. Xu, C.-H. Li, C. Xue, *Appl. Catal. B* **2015**,*169*, 572.

[5] T. Toyao, M. Saito, Y. Horiuchi, K. Mochizuki, M. Iwata, H. Higashimura, M. Matsuoka, *Catal. Sci. Technol.* **2013**, *3*, 2092.

[6] L. Shen, M. Luo, L. Huang, P. Feng, L. Wu, *Inorg. Chem*. **2015**, *54*, 1191.

[7] Y. Horiuchi, T. Toyao,M.Saito,K.Mochizuki,M.Iwata, H. Higashimura, M. Matsuoka, *J. Phys.Chem. C* **2012**, *116*, 20848.

[8] S. Pullen, H. Fei, A. Orthaber, S. M. Cohen, S. Ott, *J. Am. Chem. Soc.* **2013**, *135*, 16997.

[9] R. Lin, L. Shen, Z. Ren, W. Wu, Y. Tan, H. Fu, L. Wu, *Chem. Commun*. **2014**, *50*, 8533.

[10] A. Fateeva, P. A. Chater, C. P. Ireland, A. A. Tahir, Y. Z. Khimyak, P. V. Wiper, M. J. Rosseinsky, *Angew. Chem. Int. Ed.* **2012**, *51*, 7440.

[11] T. Zhou, Y. Du, A. Borgna, J. Hong, Y. Wang, J. Han, R. Xu, *Energy Environ. Sci.* **2013**, *6*, 3229.

[12] Y. F. Chen, L. L. Tan, J. M. Liu, S. Qin, Z. Q. Xie, J. F. Huang, Y. W. Xu, L. M. Xiao, C. Y. Su, *Appl. Catal. B* **2017**, *206*, 426.

[13] Y. Horiuchi, T. Toyao, K. Miyahara, L. Zakary, D. D. Van, Y. Kamata, T. H. Kim, S. W. Leed, M. Matsuoka, *Chem. Commun*. **2016**, *52*, 5190.

[14] Z. Lionet, T.-H. Kim, Y. Horiuchi, S. W. Lee, M. Matsuoka, *J. Phys. Chem. C* **2019**, *123*, 27501.

[15] L.-L. Qu, J. Wang, T.-Y Xu, Q.-Y. Chen, J.-H. Chen, C.-J. Shi, *Sustain. Energ. Fuels*, **2018**, *2*, 2109.

[16] G. Z. Wang, Q. L. Sun, Y. Y. Liu, B. B. Huang, Y. Dai, X. Y. Zhang, X. Y. Qin, *Chem. Eur. J.* **2015**, *21*, 2364.
